# Compound Inflorescence (S) represses fruit growth and seed development in tomato

**DOI:** 10.1186/s43897-025-00183-x

**Published:** 2026-01-08

**Authors:** Lin Weng, Li Wang, Enbai Zhou, Guixiang Wang, Xiaoyan Wang, Rong Li, Yuan Sun, Meng Li, Han Xiao

**Affiliations:** 1 National Key Laboratory of Plant Molecular Genetics, CAS Centre for Excellence in Molecular Plant Sciences, Chinese Academy of Sciences, 300 Fenglin Rd, Shanghai, 200032 China; 2 University of the Chinese Academy of Sciences, 19 A Yuquanlu, Beijing, 100049 China; 3 Research Center for Life Sciences Computing, Zhejiang Laboratory, Hangzhou, China

**Keywords:** WOX, Transcription factor, Seed development, Fruit, Tomato

## Abstract

In most fruit-bearing plants, the growth and ripening of the fruit are highly coordinated with its seeds. In tomato (*Solanum lycopersicum*), fruit growth is regulated by a number of transcription factors. The WUSCHEL-RELATED HOMEOBOX (WOX) transcription factor COMPOUND INFLORESCENCE (S)/SlWOX9 is a key regulator of inflorescence branching and embryogenesis. In this study, we show that S activity is indispensable for fruit and seed development. Ectopic *S* expression led to a decrease in seed weight and markedly suppressed endoreduplication-associated fruit growth. Conversely, the *s-classic* allele promoted fruit growth in the small-fruited *S. pimpinellifolium* accession LA1781 though the promotion was offset by fruit overload and vegetative reversion of flowers in large-fruited cultivars. Through transcriptome profiling and DNA affinity purification sequencing (DAP-seq), we discovered that S targeted a set of genes involved in seed development. Moreover, we demonstrate that S regulates fruit and seed development mainly through direct transcriptional repression of the *Mei2-like* gene *SlTEL1* (*Solanum lycopersicum TERMINAL EAR-LIKE 1*). These findings uncover a new aspect of transcription regulation of fruit growth and seed development by WOX transcription factors in tomato.

**Supplementary Information:**

The online version contains supplementary material available at 10.1186/s43897-025-00183-x.

## Core

The WOX transcription factor S/SlWOX9 bound to thousands of genes in vitro and directly repressed the transcription of at least ten genes during fruit and seed development. It represses fruit growth and seed development in part through inhibiting *SlTEL1* expression.

## Gene and accession numbers

Accession numbers for the genes used in this study are: S/SlWOX9 (Solyc02g077390); SlTEL1 (Solyc05g013930). The raw reads of RNA-seq and DAP-seq in this study have been deposited to the Genome Sequence Archive (GSA) at NGDC (National Genomics Data Center) under accession number CRA021627.

## Introduction

Tomato is an important vegetable crop cultivated world-wide. Flower number and fruit set rate are two crucial yield-determining factors. Most modern tomato cultivars produce fewer than 15 flowers per inflorescence. Several MADS box genes have been shown to regulate inflorescence branching. Mutations in these genes lead to moderate increases in inflorescence branching (Soyk et al. 2017, 2019; Alonge et al. 2020; Wang et al. 2021, 2023; Jiang et al. 2022). More strikingly, mutations in *FALSIFOLA* (*FA*), *ANTHANA* (*AN*) and *COMPOUND INFLORESCENCE* (*S*) drastically increase inflorescence branching, resulting in production of massive flowers (Molinero-Rosales et al. 1999; Lippman et al. 2008). Although flower number typically exhibits a positive correlation with yield, an excessive number of flowers can induce floral abortion and hinder fruit set due to source-sink imbalance. Fruit load exerts significant impacts on fruit weight and yield, as documented in fruit tree crops (Palmer et al. 1997; Naor et al. 2008). In tomato, plants carrying a non-functional *MULTIPLE INFLORESCENCE BRANCH 2* allele exhibit increased fruit number but reduced fruit size under high ambient temperatures, indicating a negative correlation between fruit load and fruit weight (Sun et al. 2024). For high-yield breeding, selecting lines with moderately enhanced flower production while maintaining source-sink balance is critical. Early tomato breeders preferentially selected the natural *s* allele (*s-classic*) harboring missense mutations for its promotion of large inflorescences (Lippman et al. 2008). Previous studies have further revealed that the *s* allele has a dosage effect, enabling the induction of weak inflorescence branching phenotypes, which presents promising potential for improving tomato yield (Soyk et al. 2017).

*S* encodes SlWOX9, a member of the WUSCHEL-LIKE HOMEOBOX (WOX) transcription factor family (Lippman et al. 2008). WOX proteins have been classed into three clades: ancient, intermediate and WUS (van der Graaff et al. 2009). The intermediate clade members *WOX8/9* primarily regulate embryo development. Loss-of-function mutations in *WOX9* lead to embryonic defects and disrupt meristem growth and maintenance in Arabidopsis (Wu et al. 2005, 2007). The rice (*Oryza sativa*) *WOX9* gene *OsWOX9A*, initially identified as a tiller growth regulator, is also essential for embryogenesis (Wang et al. 2014; Ren et al. 2024; Tang et al. 2024). Similarly, while tomato *s* mutants are characterized by inflorescence abnormalities, genetic analyses reveal that null *s* alleles cause embryonic lethality (Hendelman et al. 2021). Notably, *WOX9* genes evolved lineage-specific functions. In *Petunia hybrida* and *Capsicum annuum*, *WOX9* regulates flower morphogenesis (Rebocho et al. 2008; Cohen et al. 2014), whereas in *Medicago truncatula* and *Nicotiana sylvestris*, it controls leaf blade outgrowth (Lin et al. 2013; Wolabu et al. 2021; Wang et al. 2022b). Like WUS, WOX9 proteins harbor a DNA-binding homeodomain (Dolzblasz et al. 2016), yet its binding motifs and target genes remain poorly characterized (O’Malley et al. 2016; Chen et al. 2021). For instance, *Phaseolus vulgaris* and *Phaseolus coccineus* WOX9 proteins recognize a cis-element similar to Dof transcription factor binding sites (Chen et al. 2021), while *N. sylvestris* WOX9 recognizes an uncharacterized motif in the *cytokinin oxidase 3* (*NsCKX3*) promoter (Wang et al. 2022b). Given the phenotypic diversity of *wox9* mutants across species, identifying WOX9 binding sites and target genes in model systems represents a critical step toward decoding the evolutionary trajectories of WOX9-mediated regulatory networks.

Plant *TERMINAL EAR1-like* (*TEL*) genes encode Mei2-like RNA-binding proteins (Jeffares et al. 2004). While their biochemical functions remain uncharacterized and only monocot mutants have been studied, maize and rice *tel* mutants exhibit defects in leaf initiation and development (Veit et al. 1998; Kawakatsu et al. 2006; Xiong et al. 2006). Maize *tel* mutant (*zmte1-2*) displays smaller seeds, along with previously observed leaf phenotypes and tassel feminization, and shows that TE1 can be phosphorylated by WEE1 kinase, a conserved regulator of cell cycle progression (Wang et al. 2022a). Given WEE1 regulates endoreduplication in maize endosperm and tomato fruit (Sun et al. 1999; Gonzalez et al. 2007), TELs likely modulate endoreduplication-associated fruit and seed development.

Developing seeds are critical for fruit growth, supplying phytohormones like auxin and gibberellins (Dorcey et al. 2009; Ruan et al. 2012; Robert 2019). Fruit size generally correlates with seed number: reducing viable seeds in tomato decreases fruit weight, possibly via impaired cell division and expansion (Tran et al. 2023), while Arabidopsis silique length correlates with seed number (Ripoll et al. 2019). Given that *S* regulates embryo development, we hypothesized it influences fruit growth. Here, we characterized *S* functions in tomato seed development and fruit growth. Results show S acts as a transcription repressor, regulating in these processes. Through gene expression profiling and DNA–protein interaction analyses, we identified a set of S target genes during early fruit and seed development, and demonstrated that *S* regulates embryo development partially via the *Mei2-like* gene *SlTEL1*.

## Results

### *S* is a negative regulator of fruit growth

**Fig. 1:**
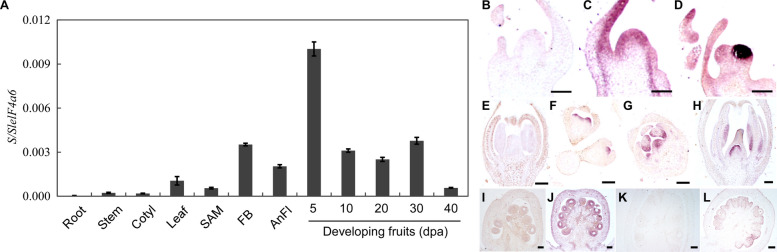
*S* expression in various tissues. **A** Quantification of *S* expression in different tissues of wild type LA1781 by qRT-PCR. *S* expression relative to *SleIF4α6* was determined in roots, stems, cotyledons (cotyl), leaves, shoot meristems (SAM), young flower buds (FB), anthesis flowers (AnFl) and developing fruits at 5-, 10-, 20-, 30- and 40-days post anthesis (DPA). Data represent means ± standard deviations (SD) of three biological replicates. Total RNA of each replicate was isolated from 5–10 fruits collected from five plants or 15–20 seedlings for vegetative tissues, respectively. **B-D** *S* expression in SAM (**B, C**) and flower meristems (**D**) of wild type LA1781 revealed by in situ hybridization. **E–H** *S* expression in flowers of LA1781 at different developmental stages revealed by in situ hybridization. **I-L** *S* expression in 5 (**I, J**) and 10 (**K, L**) DPA fruits of LA1781 revealed by in situ hybridization. Sections in (**B, E, I** and **K**) were probed by the *S* sense riboprobe. Scale bar, 100 μm

We investigated *S* expression in vegetative and reproductive tissues of wild-type *Solanum pimpinellifolium* (LA1781). Quantitative real-time polymerase chain reaction(qRT-PCR) analysis showed that *S* was expressed in leaves, shoot apical meristems (SAMs), flowers and fruits, with highest expression in the fruits at five days post anthesis (DPA) (Fig. 1A). In situ hybridization confirmed high *S* expression in SAMs, developing flowers and fruits, and further revealed its specific expression in ovules of pre-anthesis flowers and seeds after fertilization (Fig. 1B-L). Low *S* expression was detected in pericarps, septa, placenta and vascular tissues at 5 DPA (Fig. 1I, J). This spatiotemporal expression suggests that *S* functions in fruit development beyond inflorescence branching regulation.

**Fig. 2:**
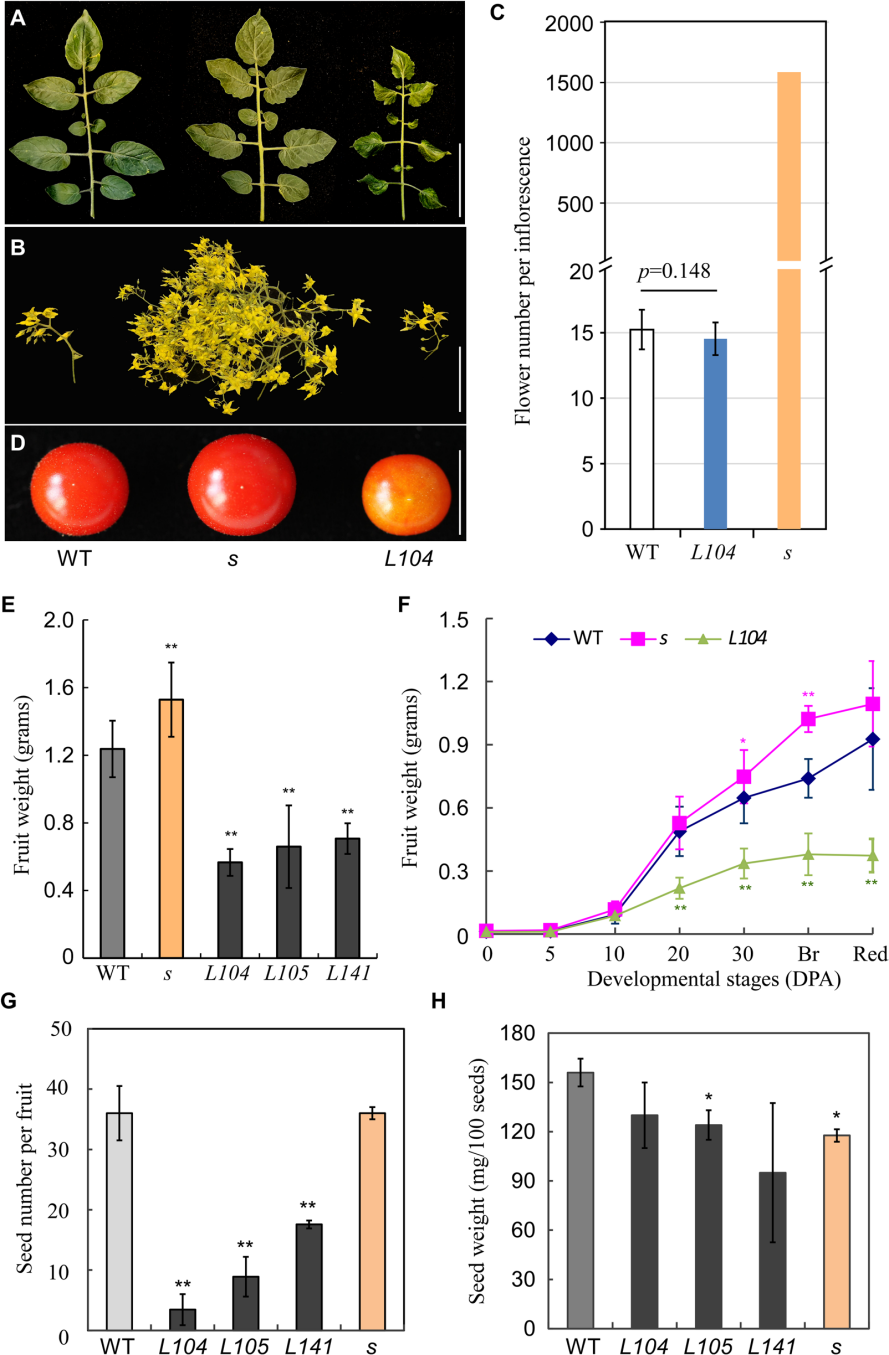
Leaf, inflorescence and fruit phenotypes of *s, S*^*OE*^ and wild type in LA1781 background. **A** Representative leaves of *s*, *S*^*OE*^ and wild type LA1781. **B** Representative inflorescences of *s*, *S*^*OE*^ and wild type. **C** Flower numbers per inflorescence of *s*, *S*^*OE*^ and wild type. *s* inflorescence had more than 1,500 flowers. **D** Representative fruits of *s*, *S*^*OE*^ and wild type. **E** Fruit weight of *s*, *S*^*OE*^ and wild type. *L104*, *L105* and *L141* were three independent *S* overexpression lines. **F** Time course analysis of fruit growth for* s*, *S*^*OE*^ and wild type. **G** Seed number of *s*, *S*^*OE*^ and wild type. **H** Seed weight of *s*, *S*^*OE*^ and wild type. Measurements of flower number, fruit weight, seed number and seed weight were conducted on five plants per genotype (N = 5). To determine flower numbers per inflorescence, the flowers on the 3rd to 5.^th^ inflorescences were counted. For the time course analysis of fruit growth, 10–23 fruits were weighed for each timepoint (N = 10–23). Seeds extracted from 8–10 fruits were counted and 100 seeds were weighted, then seed number per fruit was calculated for each plant. Data in (**D-H**) represent means ± standard deviations (SD). Statistical significance was based on Student’s t-test. *, *p* < 0.05; **, *p* < 0.01. Scale bars represent 5 (**A, B**) and 1 cm (**D**)

To evaluate the role of *S* in fruit growth, we generated CaMV35S-driven overexpression lines (*S*^*OE*^) and the *s* mutant (carrying the *s-classic* G366A allele from cv. 2–005) in LA1781, backcrossed > 5 generations for genetic uniformity. Ectopic *S* expression inhibited vegetative growth: *S*^*OE*^ lines exhibited shorter roots, smaller leaves with pointed leaflets, and reduced plant heights (Fig. 2A and Figure S1A-C**)**. Further cell morphology examination revealed that the smaller *S*^*OE*^ leaves were caused by decreased cell size (Figure S1E-I). *S*^*OE*^ plants flowered much earlier (first inflorescence at 5–6 leaves vs. 12 in wild type) and produced smaller flowers without altering inflorescence branching (Fig. 2A, B and Figure S1A-D). Conversely, the *s* mutant had slightly longer roots and larger leaf cells, in addition to previously observed phenotypes of late flowering and increased inflorescence branching (Fig. 2B, C and Figure S1C-D) (Lippman et al. 2008).

Compared to wild type, the fruits of the three *S*^*OE*^ lines were smaller, while *s* fruits were larger (Fig. 2D, E). Dynamic fruit growth monitoring showed that starting from 10 DPA apparent differences in fruit weight were observed among wild type, *s* and the *S*^*OE*^ line *L104*, with *S*^*OE*^ fruits being smallest and slowest-growing and *s* fruits being the largest and fastest-growing (Fig. 2F). *S*^*OE*^ fruits generally contained fewer, lighter seeds, though seed weight reduction only reached statistical significance in one line (*L105*), whereas the *s* mutant displayed reduced seed weight with seed number comparable to wild type (Fig. 2G, H).

**Fig. 3:**
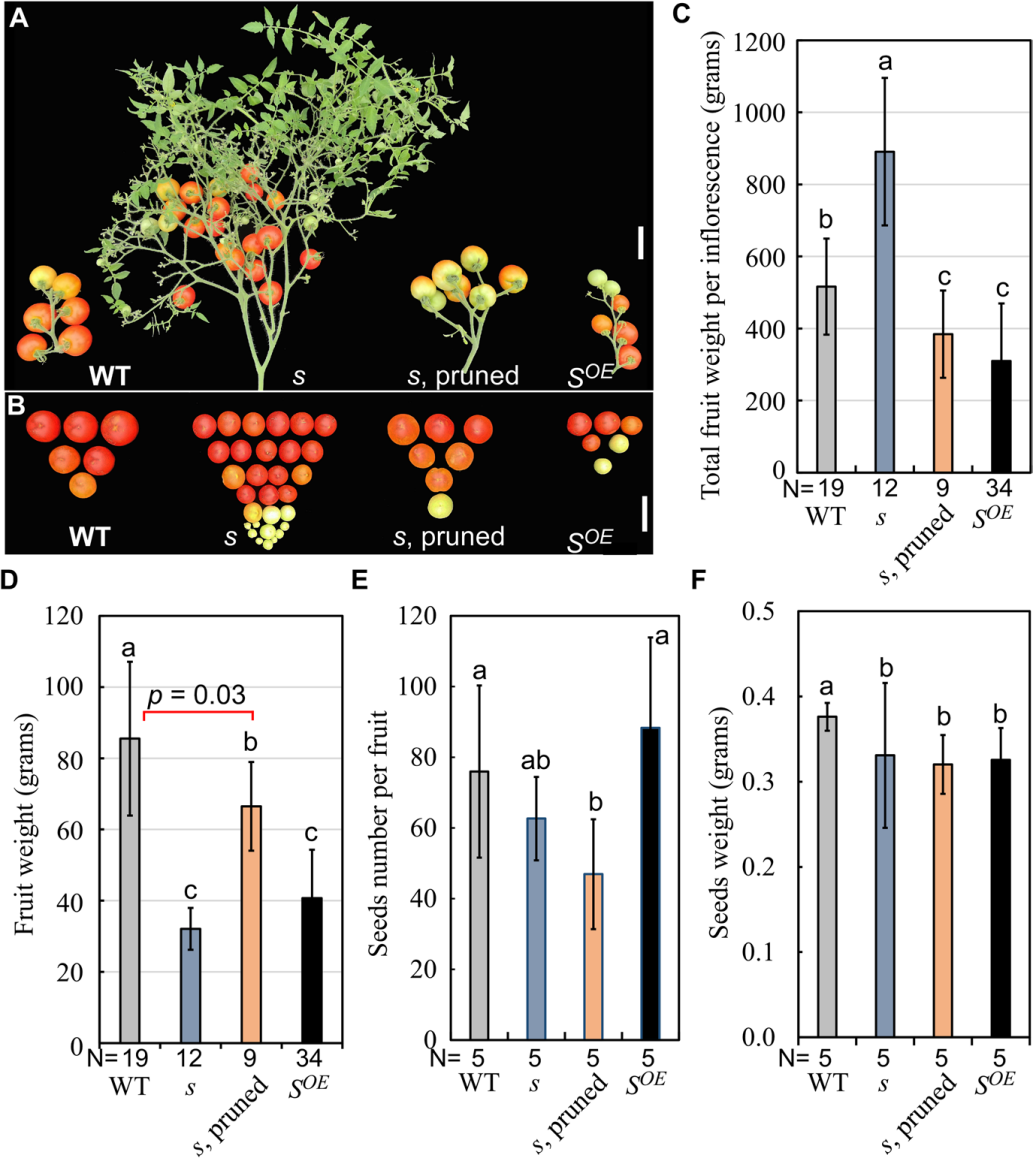
Fruit phenotypes of *s, S*^*OE*^ and wild type in Moneymaker background. **A**, **B** Fruits set on individual inflorescences of *s*, *S*^*OE*^ and wild type Moneymaker. To test whether fruit overload had impact on fruit growth, excessive flowers on the *s* inflorescences were reduced to seven flowers per inflorescence (*s*, pruned). **C** Total fruit mass per inflorescence of *s*, *S*^*OE*^ and wild type. **D-F** Fruit weight (**D**), seed number (**E**) and seed weight (**F**) of *s*, *S*^*OE*^ and wild type. Red ripen fruits were collected and weighted from 9–34 plants per genotype. For seed number and seed weight measurements, seeds were extracted from 10 fruits per plant were analyzed for each genotype (*N* = 5). Statistical significance was based on one-way ANOVA. Means sharing the same letter indicates no significant difference statistically. Scale bar, 5 cm

We further assessed the functions of the *S* gene in large-fruited tomatoes by introgressing *s-classic* and *L104* transgenes into cv. Moneymaker (LA2706). In Moneymaker, ectopic *S* expression had similarly reduced fruit and seed weight without affecting seed number (Fig. 3). The Moneymaker *s* plants exhibited strong floral vegetative reversion, increasing fruit production per inflorescence but reducing fruit weight (Fig. 3A-C). Flower thinning (7 flowers/inflorescence) alleviated fruit weight reduction without impacting seed parameters (Fig. 3D-F), indicating that fruit overload and/or vegetative reversion underlie fruit weight reduction in the Moneymaker *s* mutant. Collectively, these results show that ectopic *S* expression inhibits fruit growth, while the *s-classic* allele’s fruit-growth-promoting potential is dependent on genetic background and is compromised by fruit overload in large-fruited tomatoes.

### *S* represses endoreduplication during fruit development

**Fig. 4:**
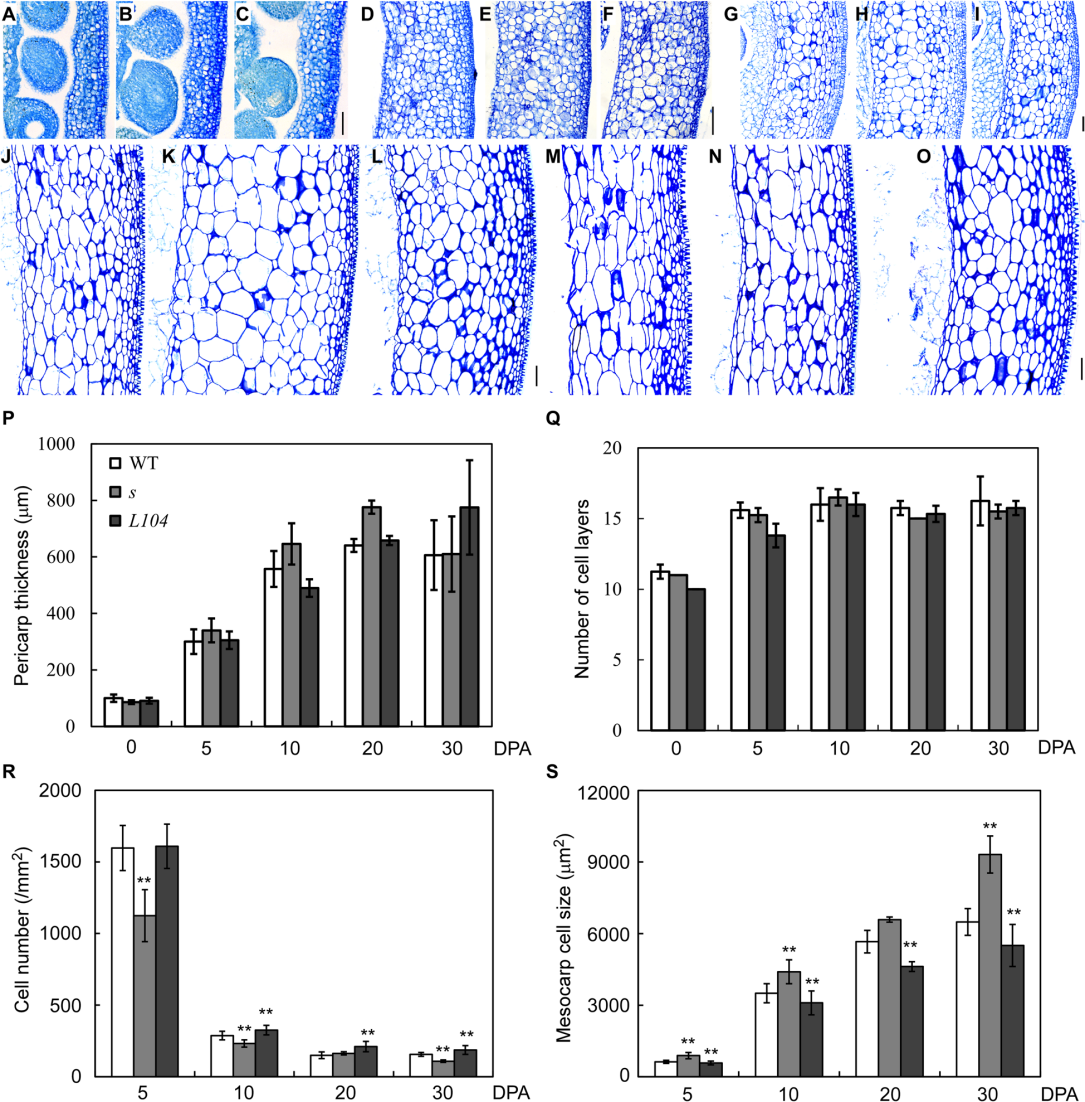
Pericarp cell morphology of *s*, *S*^*OE*^ and wild type in LA1781 background. **A-O** Sections of 0 (**A-C**), 5 (**D-F**), 10 (**G-I**), 20 (**J-L**) and 30 (**M–O**) DPA fruit pericarps of wild type (**A, D, G, J, M**), *s* (**B, E, H, K, N**) and *S*^*OE*^ line L104 (**C, F, I, L, O**). **P** A time course analysis of pericarp thickness of *s*, *S*^*OE*^ and wild type. **Q** Time course analysis of pericarp cell layers of *s*, *S*^*OE*^ and wild type. **R** Time course analysis of pericarp cell numbers of *s*, *S*^*OE*^ and wild type. **S** Time course analysis of mesocarp cell size of *s*, *S*^*OE*^ and wild type. Data in (**P-S**) represent means ± standard deviations (SD) from three biological replicates (N = 3). **, *p* < 0.001 based on Student’s t-test. Scale bar, 100 μm

Pericarp expansion is tightly linked to fruit growth in tomato (Gonzalez et al. 2007; Zhao et al. 2021). To better understand how *S* regulates fruit growth, we analyzed dynamic changes of cell division and expansion in the pericarps of wild type, *s* and *S*^*OE*^ (*L104*) in LA1781. At anthesis, pericarp thickness did not show difference among the three genotypes (Fig. 4A-C, P). From 5 to 20 DPA, *s* mutant pericarps were thicker, while *S*^*OE*^ pericarps were thinner, compared to wild type (Fig. 4D-L, P). By 30 DPA (breaker stage), thickness differences diminished (Fig. 4M-O, P). Cell layer counting from epidermis to endocarp was not severely affected by the overexpression of and mutation in the *S* gene, except slightly fewer layers in *S*^*OE*^ at early stages (anthesis to 5 DPA; Fig. 4Q), indicating *S* likely plays a minor role in regulation of cell division. We then measured mesocarp cell sizes of *s*, *S*^*OE*^ and wild type fruits at different stages. Compared to wild type, *s* mesocarp cells were larger from 5 to 30 DPA, while those of *S*^*OE*^ were smaller (Fig. 4R, S). These results suggest *S* regulates pericarp cell expansion.

**Fig. 5:**
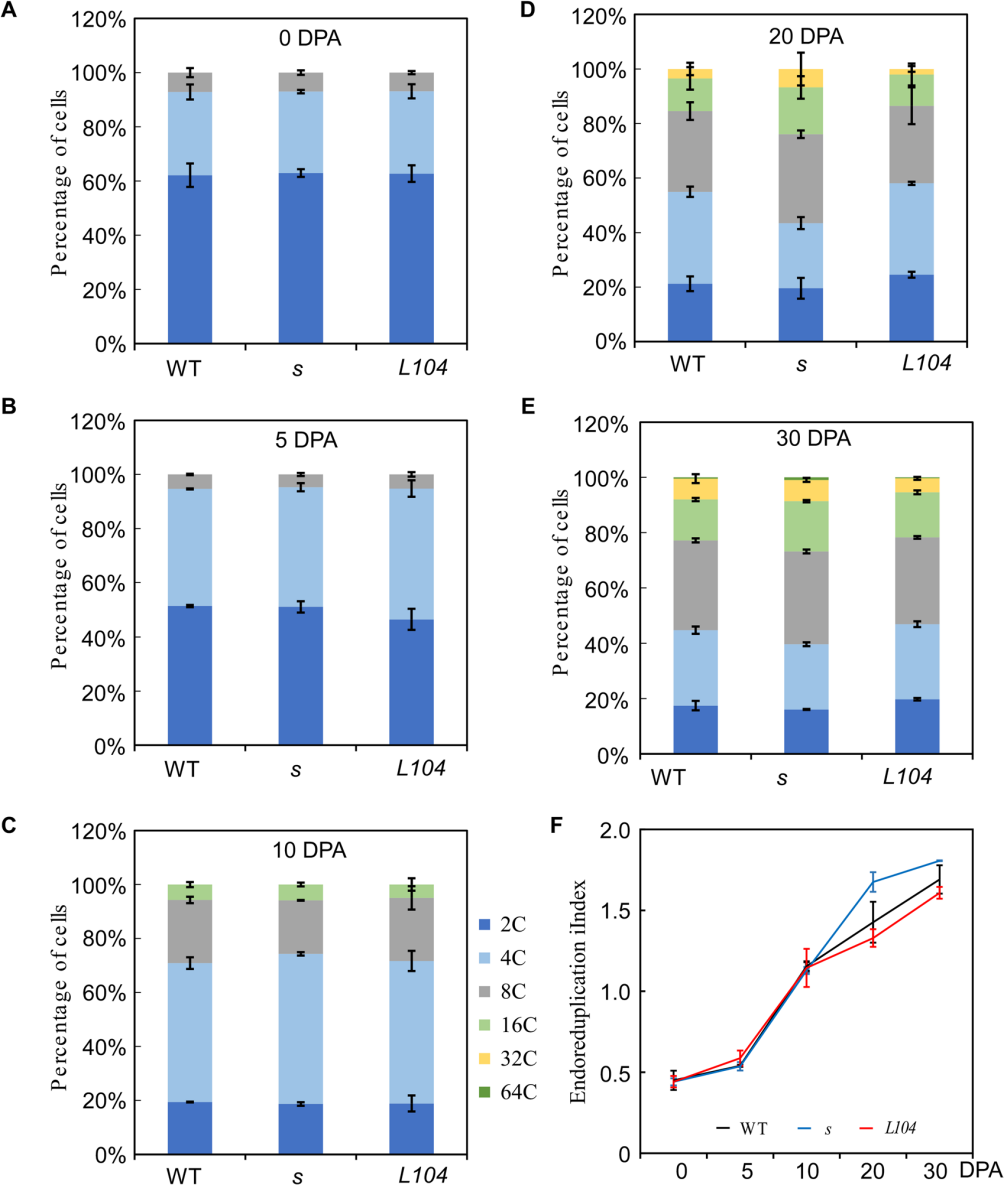
DNA ploidy level of pericarp cells of *s*, *S*^*OE*^ and wild type in LA1781 background. **A-E** Distribution of pericarp cells of different ploidy levels at anthesis (**A**), 5 (**B**), 10 (**C**), 20 (**D**) and 30 (**E**) dpa. **F** Dynamic changes of endoreduplication index in developing pericarps. The experiments were repeated on two different batches of plants with three biological replicates

In tomato, pericarp cells undergo substantial endoreduplication, a process associated with nuclei volume increase and cell enlargement (Sugimoto-Shirasu and Roberts 2003; Chevalier et al. 2014; Tourdot et al. 2023). By monitoring DNA ploidy levels of pericarp cells using flow cytometry, we found that *S* regulates endoreduplication during fruit expansion. Before 5 DPA, more than 90% of pericarp cells were 2 C and 4 C, with rare 8 C cells, and no significant difference in DNA ploidy levels was detected (Fig. 5A, B). From 10 DPA onward, ploidy levels increased differentially: the percentages of 8C-64C cells were increased faster in *s* pericarps, while *S*^*OE*^ pericarps showed slower ploidy elevation, relative to wild type. By 30 DPA, *s* mutant pericarps had the highest percentages of 16 C and 32 C cells, and *S*^*OE*^ had the lowest (Fig. 5C-E). Using endoreduplication index (EI) to evaluate the status of endoreduplication, we confirmed *S* modulates endoreduplication dynamics: EIs did not differ before 10 DPA but diverged at 20 DPA, with *s* mutant having higher EIs and *S*^*OE*^ lower EIs than wild type (Fig. 5F). Collectively, these data demonstrate that *S* suppresses endoreduplication in pericarp cells during fruit development.

### Transcriptional regulation of fruit growth mediated by the *S* gene

**Fig. 6:**
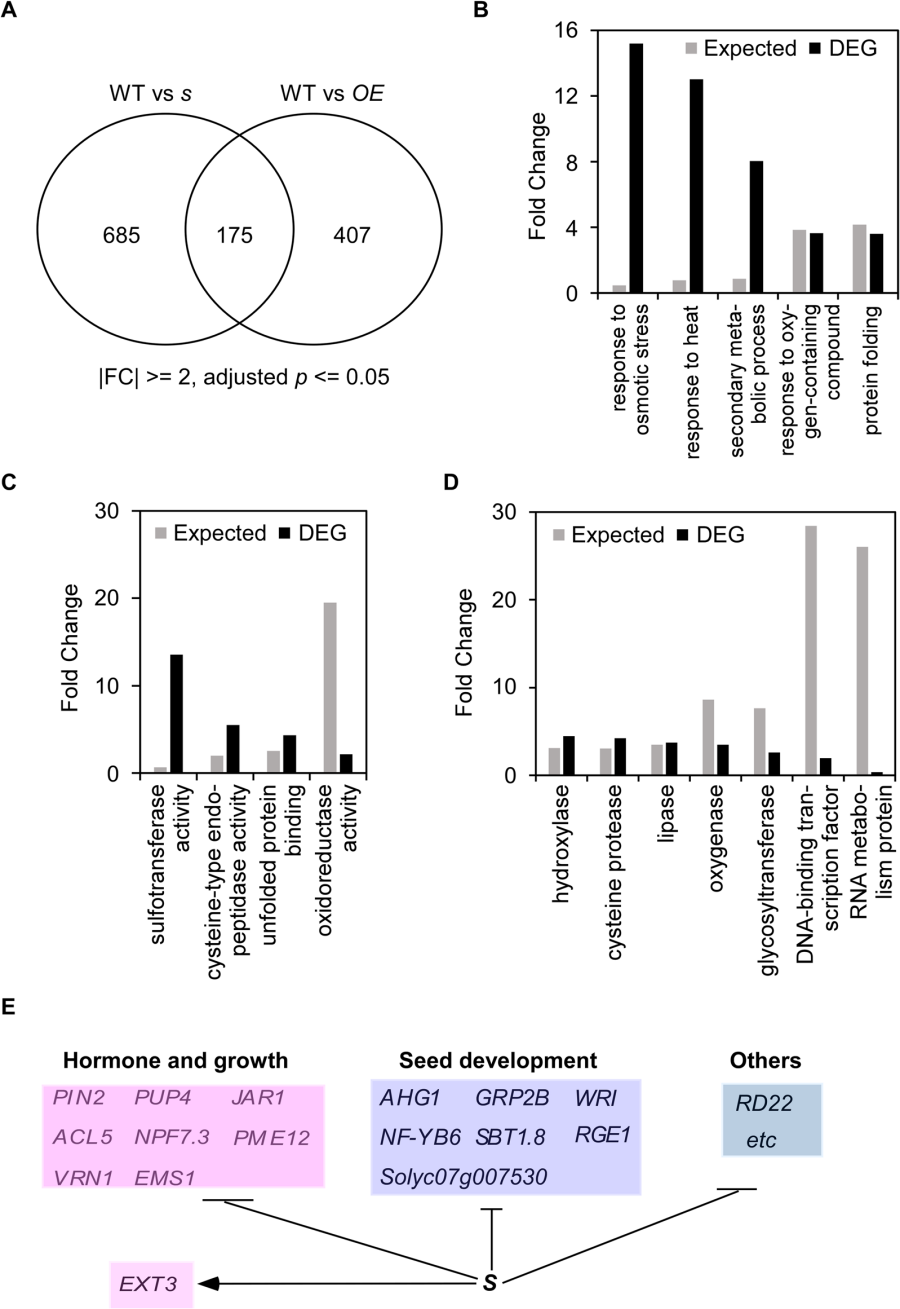
Identification of S-regulated genes in 10 DPA fruits. **A** Van diagram showing differentially expressed genes (DEGs) identified in the 10 DPA fruits of the *s* mutant and *S*^*OE*^ (*L104*). **B-D** Enriched categories of biological processes (**B**), molecular functions (**C**) and protein classes (**D**) in these DEGs. Go-slim terms analysis was conducted on the 1267 DEGs using PANTHER. Only significantly enriched terms (Bonferroni-corrected for P < 0.05 and fold enrichment larger than two-fold) were shown here. **E** *S-*regulated genes involved in hormone pathway, plant growth and seed development

Because *S* mainly regulates cell expansion, we performed an RNA-seq analysis on 10 DPA fruits, when cell division is nearly completed and cell expansion has begun, to identify *S*-regulated genes involved in cell expansion. Differentially expressed genes (DEGs) were identified using following criteria: more than two-fold changes and adjusted *p*-values less than 0.05. In total, 1,267 DEGs were identified (Fig. 6a; Table S1), of which 860 and 582 genes were differentially expressed in *s* and *S*^*OE*^ (*L104*) fruits, respectively (Table S2 and S3). Gene ontology (GO) analysis using PANTHER revealed that several GO-slim terms for biological processes were highly enriched, including “response to osmotic stress”, “response to heat”, and “secondary metabolic process” (Fig. 6B). Molecular function categories like “sulfotransferase activity”, “cysteine-type endopeptidase activity”, “unfolded protein binding” and “oxidoreductase activity” were enriched (Fig. 6C). DNA-binding transcription factor was one of the enriched protein classes (Fig. 6D). Cell cycle-related genes were not significantly enriched, implying that *S* might not directly regulate cell cycle during fruit development. However, the expression of several cell cycle genes was altered in either *s* mutant or *S*^*OE*^ fruits at 10 DPA. Specifically, three cyclin genes (*Solyc04g081650, Solyc04g081655* and *Solyc04g081660/CYCB2_3*) were only expressed in *S*^*OE*^ fruits.

We further identified putative *S*-regulated genes during fruit development. *S*-regulated genes were defined by reciprocal expression changes: repressed in *s* fruits but upregulated in *S*^*OE*^, or vice versa, along with genes exclusively expressed in *s* or *S*^*OE*^ fruits. In total, 109 DEGs were putative *S*-regulated genes (Table S4). Among these, several are likely involved in hormone pathways and plant growth, including *PIN-FORMED 2* (*PIN2*), *ACAULIS 5* (*ACL5*), *JASMONATE RESISTANT 1* (*JAR1*), *PECTIN METHYLESTERASE 12* (*PME12*), *NRT1/PTR FAMILY 7.3* (*NPF7.3*) and *PURINE PERMEASE 4* (*PUP4*) (Fig. 6E; Table S4). Another subset are homologs of Arabidopsis genes known for their roles in regulation of seed development, such as *ABA-hypersensitive germination 1* (*AHG1*), *Nuclear transcription factor Y subunit B6* (*NF-YB6*), *WRINKLED 1* (*WRI1*), *Glycine-rich protein 2B* (*GRP2B*), *SUBTILASE 1.8* (*SBT1.8*), *RETARDED GROWTH OF EMBRYO 1* (*RGE1*) and *CYCLOPS 1* (*CYL1*). Strikingly, among these, only *EXTENSIN 3* (*ETX3*) was positively regulated by *S*, whereas the remaining 97 genes (including 11 *S*^*OE*^-exclusive genes) were transcriptionally repressed by *S* (Fig. 6E; Table S4).

### Identification of direct S targets during fruit development

**Fig. 7:**
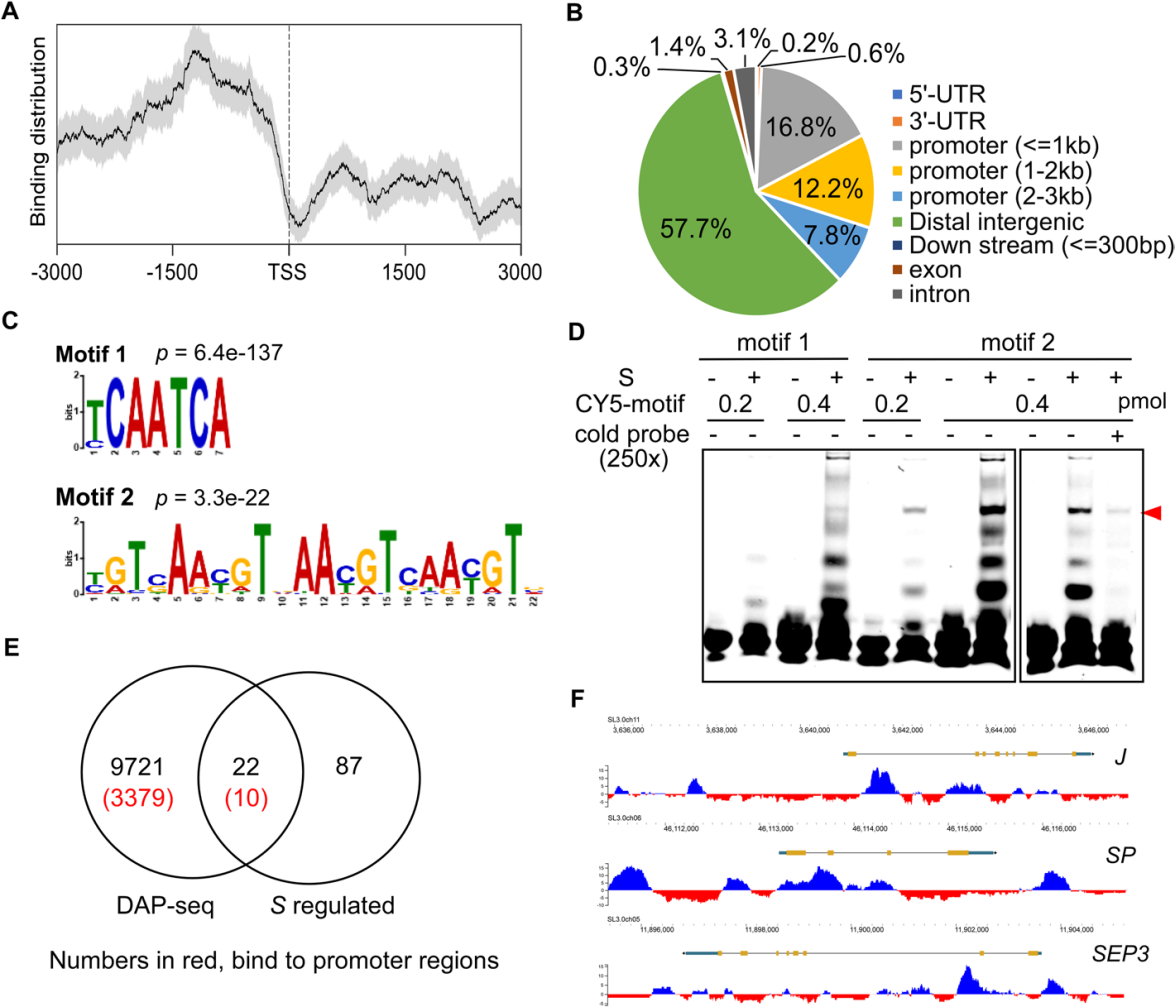
Identification of S binding sites. **A** Distribution of S-bound sequences on genic regions. **B** Genomic location of S binding sites along its target genes. **C** Enriched S binding motifs identified by MEME-ChIP. **D** Verification of the binding affinity between S and the two motifs (motif 1 and 2) by EMSA. Cold probe, unlabeled probe as competitor. The red arrowhead indicates the shifted bands containing protein and probe. **E** Van diagram showing the number of S-regulated and S-bound genes. The number of genes containing S binding sites on the promoter regions are highlighted in red. **F** Gbrower snapshots showing S binding on the genomic regions of flowering regulators *J*, *SP* and *SEP3*

To identify the genes directly regulated by *S*, we conducted DNA affinity purification sequencing (DAP-seq) to map S-bound chromatin regions. Mapped reads were enriched within 1.0–1.5 kb upstream of predicted transcription start sites (TSS), with 37% of bound sequences located within 3 kb of start codons, and 57.7% in intergenic regions (Fig. 7A, B). In total, DAP-seq identified 9,721 S-bound regions, including 3,379 in promoter regions (Table S5). Motif analysis uncovered two consensus sequences: Motif 1 (T/C)CAATCA and motif 2 [T(C/G)AA(C/T)GT], a three-repeat imperfect sequences (Fig. 7C). Motif 1 shares homology with WUS binding motif identified by DAP-seq (O’Malley et al. 2016). Electrophoretic mobility shift assay (EMSA) confirmed strong S binding to motif 2 and weaker binding to motif 1 (Fig. 7D).

By comparing DAP-seq and RNA-seq data, we identified ten putative S target genes, defined by being S-regulated genes with binding sites within 2 kb of their promoters (Fig. 7E). Among these, *ACL5* and *Solyc11g051090* (*sulfotransferase 12*) are implicated in fruit growth. *Solyc09g083310*, whose Arabidopsis homolog *DREB2A-INTERACTING PROTEIN 1* that controls carpel development (Chen et al. 2016), may also participate in fruit development. *PME12, NF-YB6* and the egg cell-secreted-like gene *Solyc07g007530* are associated with seed development. The remaining four S targets (*Solyc02g062310, Solyc02g062320*, *Solyc02g070170* and *Solyc07g053300*) have uncharacterized roles in fruit growth and seed development.

*S* positively regulates flowering, as evidenced by delayed and accelerated flowering phenotypes in *s* and *S*^*OE*^ plants, respectively. Using a stage-by-stage SAM transcriptome data (Park et al. 2012), we examined S binding sites in genes differentially expressed during the vegetative-reproductive transition between wild type and the *s* mutant. In total, we found S bound to the promoter regions of 431 DEGs, including key flowering regulators: *UNIFLORA* (*UF*), *JOINTLESS* (*J*), *SELF-PRUNING* (*SP*) and *SEPALLATA* (*SEP3*) (Fig. 7F; Table S6).

### *S* represses *SlTEL1* to orchestrate fruit growth and seed development

Gene co-expression analysis has been employed to uncover gene regulatory relationships (Li et al. 2023). Given the post-fertilization *S* expression in fruit tissues (Fig. 1), we performed co-expression analysis to identify candidate genes collaborating with *S* in fruit development. Using fruit tissue-specific transcriptome data, we identified *Solyc05g013930* as the top co-expressed gene (correlation coefficient *r* = 0.78) (Shinozaki et al. 2018). *Solyc05g013930,* designated here as *SlTEL1*, this gene encodes a Mei2-like RNA-binding protein with highest sequence similarity to Arabidopsis AtTEL1, orthologous to maize TE1 (Figure S2). Notably, maize *te1* mutants exhibit developmental abnormalities, including reduced seed size, prompting us to hypothesize that *SlTEL1* acts as a critical mediator of S-driven regulation in tomato fruit and seed development.

**Fig. 8:**
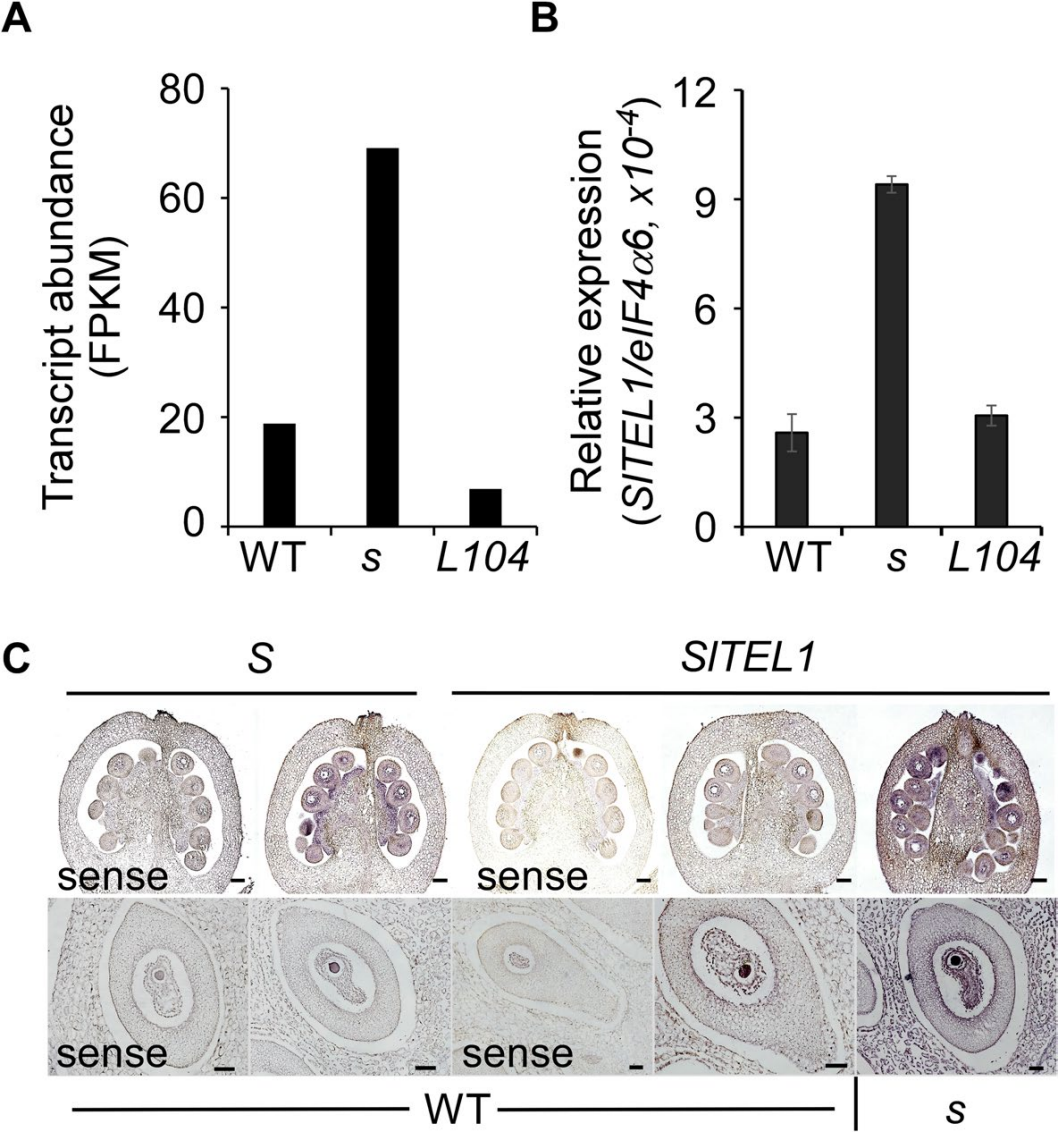
*S* negatively regulates *SlTEL1* expression in developing fruits. **A** Transcript abundance of *SlTEL1* in the 10 DPA fruits of wild type, *s* and *S*^*OE*^ (*L104*) detected by RNA-seq. RPKM, reads per kilobase of million mapped reads. **B** Quantification of *SlTEL1* expression by qRT-PCR in the 10 DPA fruits of wild type, *s* and *S*^*OE*^ (*L104*)*.* The data are presented as mean ± standard deviation from three biological replicates. **C** *SlTEL1* expression in the fruits (2 DPA) and seeds (10 DPA) of wild type and the *s* mutant. The data presented here were collected from plants in LA1781 background. Scale bar, 100 μm

The *SlTEL1* expression was respectively increased and decreased by more than two folds in the 10 DPA fruits of *s* and the *S*^*OE*^ line L104 (Fig. 8A). qRT-PCR validation using independent samples confirmed elevated expression in *s* fruits, despite its low basal expression at 10 DPA (Fig. 8B). Further in situ hybridization analysis revealed overlapping expression domains of *SlTEL1* and *S* in fruit tissues, particularly seeds, with elevated *SlTEL1* expression detected in seeds and other tissue parts of *s* fruits at 5 DPA (Fig. 8C).

**Fig. 9:**
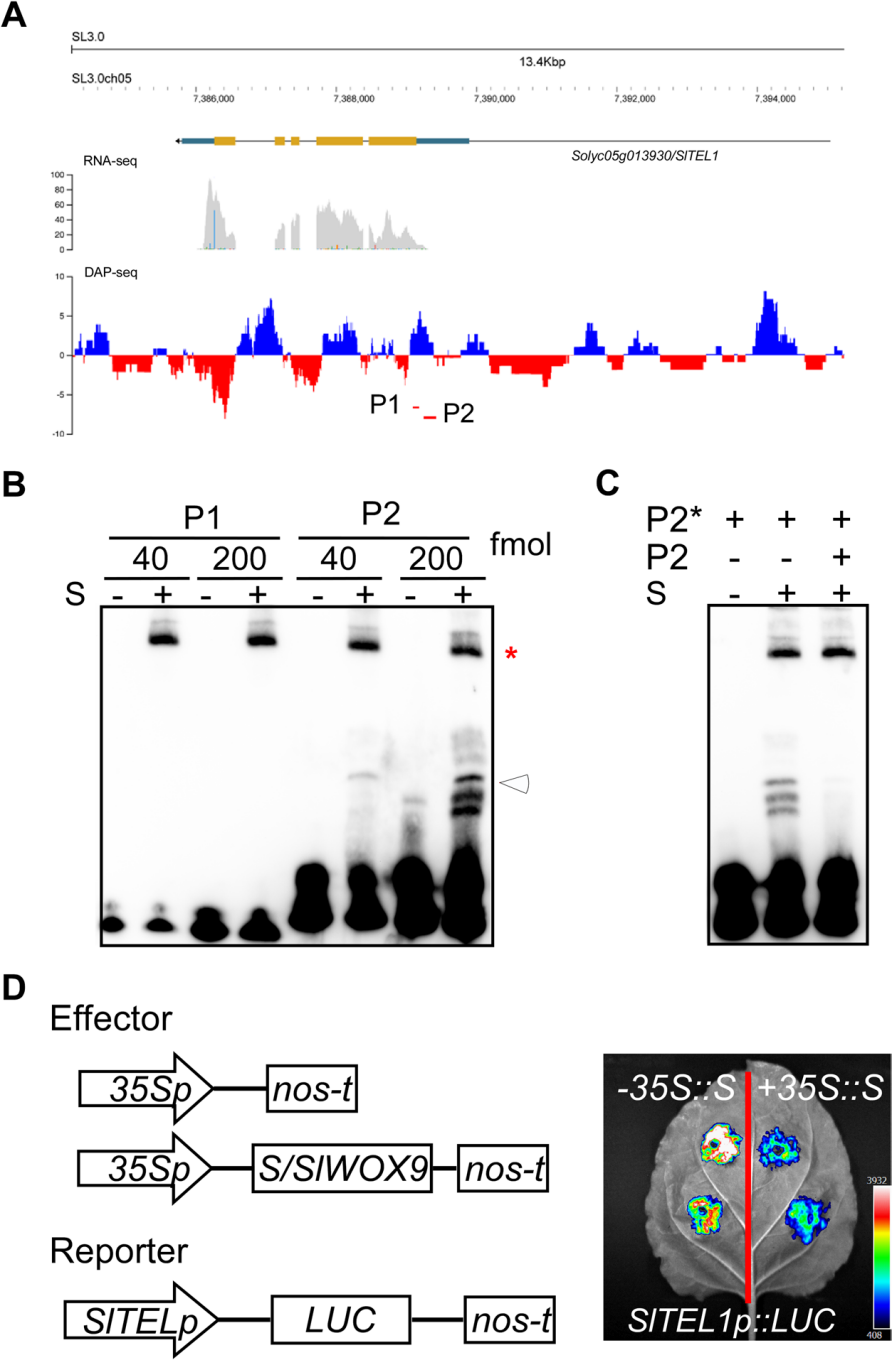
*S* directly represses *SlTEL1* transcription. **A** S bound to the promoter region of the *SlTEL1* gene. RNA-seq reads and DAP-seq reads were aligned to the tomato reference genome version ITAG3.0. P1 and P2, which contain S binding motifs, are located in the putative 5’ untranslated region (5’ UTR) of the *SlTEL1* gene. **B** S binding activity on P1 and P2 probes detected by EMSA. *, non-specific binding. The white arrowhead indicates the shifted band containing protein and probe. **C** Specificity of S binding on P2. The CY5-labeled P2 probe is depicted as P2*, the unlabeled P2 probe (250-fold) as competitor (P2). **D** Transient expression assay showing repression of *SlTEL1* by S. The reporter (*SlTEL1p::LUC*) was co-infiltrated with the effector *35S::S* or empty vector (−35S::S, control) into tobacco leaves and LUC activity was assayed two days later

Moreover, we found S bound to the proximal promoter region of *SlTEL1*, which is misannotated as an intron in the ITAG4.0 gene model (Fig. 9A). Using EMSA, we confirmed S binding to a DNA fragment containing Motif 2 in the *SlTEL1* promoter (Fig. 9B). Similarly, overexpressing S strongly repressed LUC expression driven by the 2.3 kb *SlTEL1* promoter in tobacco leaves (Fig. 9C, D). Collectively, these data demonstrate that S directly represses *SlTEL1* expression during early fruit development.

**Fig. 10:**
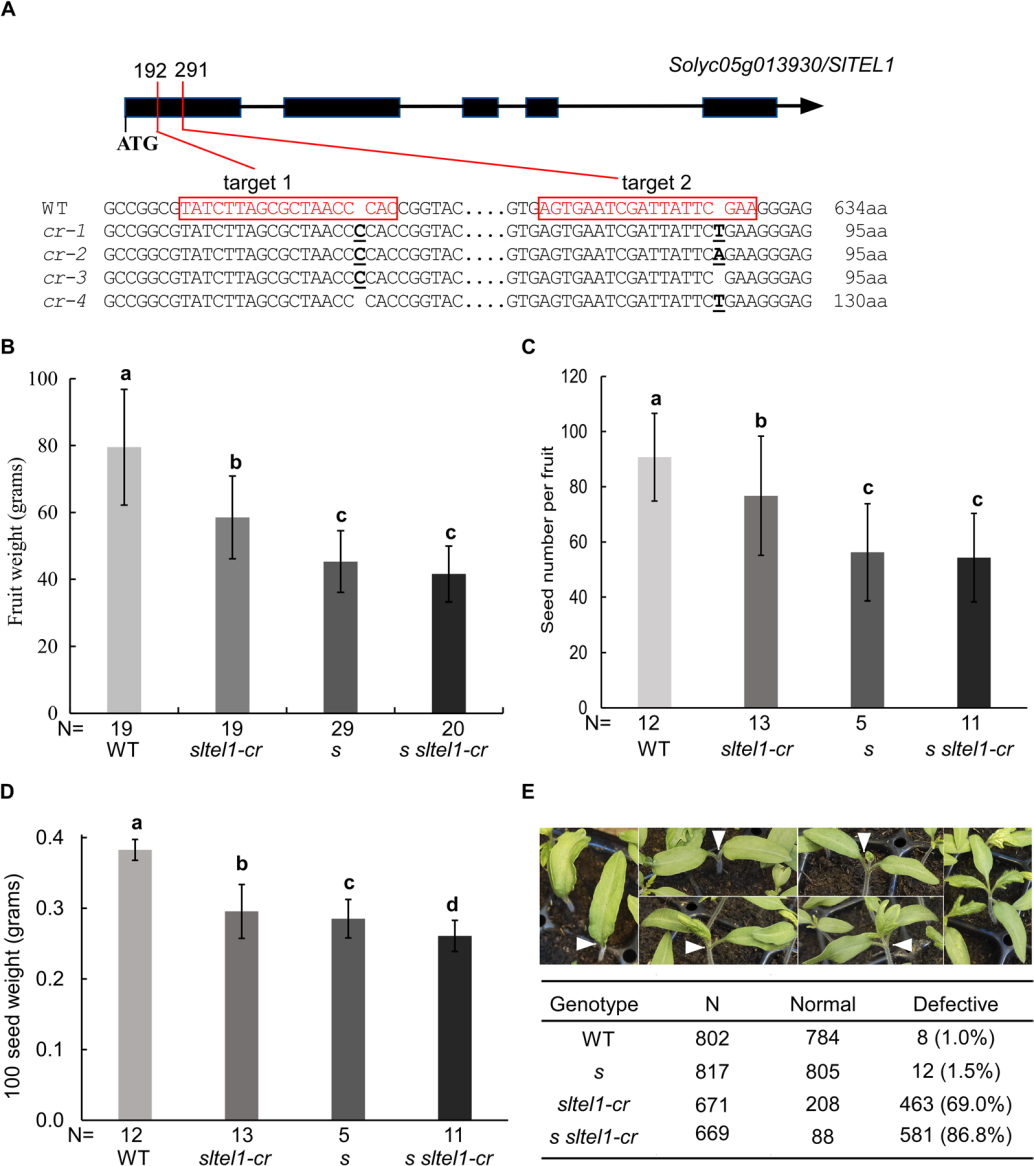
Genetic interaction between *SlTEL1* and *S* in regulation of fruit and seed development. **A** Fruit weight of wild type, *sltel1, s* and *sltel1 s*. **B** Seed number of wild type, *sltel1, s* and *sltel1 s*. **C** Seed weight of wild type, *sltel1, s* and *sltel1 s*. **D** Embryogenic defects caused by *SlTEL1* mutation. White arrowheads indicate abnormal development of cotyledons and shoot apical meristems including early arrest of shoot meristem after either none or few leaves formed. Data represent means ± standard deviations (SD). Statistical significance was based on one-way ANOVA. Means sharing the same letter indicates no significant difference statistically

To determine whether *S* regulates fruit and seed development through *SlTEL1*, we generated *sltel1* mutants in Moneymaker background by CRISPR-Cas9. Four *sltel1* mutant alleles (*sltel1-cr1* to -*cr4*) were obtained, each introducing premature stop codons after 95 or 130 amino acids (Fig. 10A). We selected *sltel1-cr1* as a representative allele to test the genetic epistasis between *S* and *SlTEL1.* To minimize negative impact of fruit overload, we limited each inflorescence fruits to 5–7 fruits. Compared to wild type, both *s* and *sltel1-cr1* produced smaller fruits with fewer and lighter seeds, but the reductions in *sltel1-cr1* were less severe (Fig. 10B-D). Fruit weight and seed number of *s sltel1-cr1* were similar with those of the single *s* mutant, suggesting that *S* likely acts upstream of *SlTEL1* in these processes. However, *s sltel1-cr1* seeds were lighter than those of either single mutant, indicating synergistic regulation of seed weight by *S* and *SlTEL1* mutations.

Further investigation of seedling morphology revealed that 69% of *sltel1-cr1* and 86.8% of *s sltel1-cr1* seedlings exhibited SAM arrest, with defects ranging from complete failure to form leaves to arrest after 1–2 leaf primordia (Fig. 10E). By contrast, only 1.0% and 1.5% of wild type and *s* mutant seedlings showed such defects. The higher frequency of defective seedlings in *s sltel1-cr1* indicates that *SlTEL1* collaborates with *S* to safeguard embryo development. These results demonstrate that S regulates fruit and seed development primarily through transcriptional repression of *SlTEL1*, while additional parallel pathways likely contribute to seed weight and embryo viability.

## Discussion

*WOX9* genes display diverse functions across plant lineages, regulating developmental processes from leaf blade outgrowth, flowering to inflorescence branching and embryo development (Wu et al. 2005; Skylar et al. 2010; Wang et al. 2014; Zhu et al. 2014; Hendelman et al. 2021; Wolabu et al. 2021; Ren et al. 2024). However, their role in fruit development has not been characterized. In this study, we demonstrated that S, the tomato WOX9 ortholog, negatively regulates fruit size through a combination of actions on cell cycle regulation in fruit tissues and seed development, which depends in part on the Mei2-like protein SlTEL1, an ortholog of maize TE1.

### *S* negatively regulates fruit growth

Fruit weight reduction in *S*^*OE*^ lines across both cultivated tomato (Moneymaker) and wild relative (*S. pimpinellifolium*LA1781) genetic backgrounds (Figs. 2 and 3) confirms *S* as a conserved negative regulator of fruit growth. Conversely, the *s-classic* allele increased fruit weight in LA1781 but failed to do so in Moneymaker, likely due to source-sink imbalance, a common challenge in large-fruited cultivars (Stephenson 1981). This imbalance was exacerbated in Moneymaker *s* mutants by massive floral reversion to vegetative tissues, a phenotype less frequent in LA1781. Flower thinning experiments supported this mechanism: reducing inflorescence flower number to 7 significantly increased fruit weight (Fig. 3), demonstrating that fruit overload mediates the negative effect of *s-classic* in large-fruited backgrounds. Another possibility we can’t rule out is that there is genetic epistasis between *S* and uncharacterized loci in large-fruited cultivars. Such interaction has been observed between *j2*^*TE*^ and *ej2*^*w*^ in controlling tomato fruit weight and inflorescence branching (Soyk et al. 2017). Moreover, the *s-classic* allele has dosage effect on inflorescence branching, enabling breeding of weakly branched lines with improved yield potential (Soyk et al. 2017). From a breeding perspective, evaluating *s-classic* in diverse genetic backgrounds—particularly elite large-fruited varieties—will be critical to uncouple inflorescence architecture from fruit growth and optimize yield components.

Fruit weight is intricately determined by cell number and size, with the former linked to mitotic duration/rate and the latter to cell expansion. In tomato, a short cell division phase upon successful fertilization precedes cell expansion phase that often spans a few weeks (Xiao et al. 2009). Our results suggest that *S* exerts a repressive role on endoduplication-associated fruit growth. Endoreduplication, a process defined by genome replication in the absence of mitotic cell division, functions as a significant mechanism governing cell growth in organs such as fruits (Chevalier et al. 2011; Zhao et al. 2021). This process leads to an increase in DNA ploidy levels, which in turn attribute to cell enlargement. Consistent with this, the *s* mutant showed enhanced endoreduplication (higher ploidy) and cell expansion, whereas *S*^*OE*^ lines exhibited reduced ploidy and cell size (Figs. 4 and 5). The *S*^*OE*^ phenotype—fewer pericarp cell layers at 0–5 DPA but normal layers at maturity—suggests *S* delays the transition from cell division to expansion. Root and leaf growth were inhibited in *S*^*OE*^ lines, a phenotype also observed in the gain-of-function mutant of *WOX9* in Arabidopsis (Wu et al. 2005). In contrast, Arabidopsis *WOX9* promotes cell cycle entry to maintain cell division during vegetative development (Wu et al 2005), while in *N. sylvestris* and *M. truncatula*, it facilitates leaf cell division and cell differentiation (Wang et al. 2022b). Rice *DWARF TILLER 1*/*OsWOX9A* positively regulates tiller cell division during tiller growth, with its mutant showing reduced internode cell number and size (Wang et al. 2014). These observations collectively imply a conserved role for WOX9 in regulating post-embryonic cell division and cell differentiation.

Arabidopsis *WOX9* promotes cell division by activating *CYCB1;1* and *CDKB1;1*, two key regulators of the G2-to-M transition (Wu et al. 2005). Our DAP-seq analysis revealed S binding to promoters of cyclin genes and cyclin-dependent kinase inhibitors, *SIAMESE-RELATED 4* and *8* (Table S4). However, these cell cycle related genes were not in the list of *S*-regulated genes, likely due to our stringent DEG selection criteria. For instance, *Solyc12g087900* (*cyclin D2_1*) was upregulated in *s* fruits and repressed in *S*^*OE*^ fruits but the repression was not statistically significant. Our transcriptome analysis conducted on whole fruits at a single timepoint may have missed spatiotemporal expression dynamics, potentially underestimating *S-*mediated regulation of cell cycle genes. For example, transient transcriptional changes in specific tissues (e.g., developing seeds or pericarp) might not be captured in bulk RNA-seq data. Thus, whether *S* directly controls cell cycle gene transcription during fruit development requires further investigation.

### *S* regulates fruit and seed development dependent on SlTEL1 activity

Our findings show that *S* controls seed development in addition to fruit growth, consistent with its reported role in embryogenesis (Hendelman et al. 2021). We demonstrate that *S* regulates seed development largely through directly repressing *SlTEL1* expression, supported by three lines of evidence: 1) co-expression of S and SlTEL1 in developing seeds during fruit growth; 2) in vitro and in vivo binding of S to the *SlTEL1* promoter to repress its expression; 3) genetic interaction analysis placing *SlTEL1* downstream of *S*. The more severe seed weight and seedling defects in the double mutant *s sltel1* suggest additional genes contribute to this pathway.

While null mutations in the *S* gene are embryo-lethal (Hendelman et al. 2021), preventing direct genetic interaction studies, *S* also regulates homologs of *WRI1, RGE1, NF-YB6* and *SBT1.8* involved in seed development (Table S3). Mutations in these genes cause developmental defects in Arabidopsis seeds (Focks and Benning 1998; Kwong et al. 2003; Kondou et al. 2008; Fiume et al. 2016). Given *tel1* and loss-of-function mutants of these genes lack embryo-lethal phenotype, *S* likely regulates seed development through multiple genetic pathways.

SlTEL1 is the tomato ortholog of maize TE1. Analyses of maize and rice *tel* mutants reveal a conserved role in leaf initiation in monocots (Kawakatsu et al. 2006; Xiong et al. 2006; Wang et al. 2022a). Unlike monocots, *sltel-cr* mutants exhibit no obvious leaf initiation defects (e.g., shortened plastochron or precocious maturation), except for SAM arrest due to embryonic abnormalities. Arabidopsis has two *TEL* genes expressed in apical meristems, but single mutants show no phenotypes, likely due to functional redundancy (Anderson et al. 2004). Given that *SlTEL1* and the two Arabidopsis *TEL* genes are expressed in embryos, *TEL* family members may play conserved roles in dicot embryo development.

Fruit weight reduction in *sltel1-cr* mutants indicates *SlTEL1* directly or indirectly regulates tomato fruit growth. Similarly, the maize *zmte1-2* nonsense mutation reduces seed size (Wang et al. 2022a). TE1 promotes seed growth likely through cell cycle regulation as it enhances the expression of cell cycle-related genes in the stems. Moreover, TE1 can be phosphorylated by maize cell cycle regulator WEE1 (Wang et al. 2022a). Given tomato *WEE1* regulate fruit size through its action on endoreduplication process (Ganzalez et al., 2007), *SlTEL1* may similarly link cell cycle control to fruit grwoth though an unchracterized mechanism. Other members of Mei2 family also regulate cell size. The rice *Mei2-like protein 4* (*OML4*)*,* a distinct Mei2 subgroup member, represses cell expansion in spikelet hull; its loss-of-function mutation increases grain size (Lyu et al 2020). In addition, *OML4* and its Arabidopsis homologs regulate flowering time through interaction with other proteins (Anderson and Hanson 2005; Cui et al. 2024), indicating *Mei2-like* genes have multiple roles in plant development. Whether seed phenotypes in these mutants reflect pleiotropy or specific developmental pathways remains unclear.

Seed development is intimately linked to fruit size. in Arabidopsis and tomato, seed number positively correlates with fruit size (Ripoll et al. 2019; Tran et al. 2023), likely because developing seeds supply hormones such as auxin and gibberellins to fruit tissues. In maize *zmte1-2*, auxin transporters and signaling genes are significantly downregulated, disrupting auxin transport from the seeds to fruit tissues (Wang et al. 2022a). Likely, loss-of-function of *SlTEL1* may compromise auxin transport, reducing fruit growth. Indeed, *S* repressed *SlPIN2* expression in 10 DPA fruits. We speculate *S*-mediated fruit growth regulation involves seed-derived auxin transport dependent on SlTEL1.Nonetheless, it is of great interest to further dissect the regulatory mechanism of fruit growth and seed development controlled by *SlTEL1*.

### Transcriptional regulation mediated by S

S binds to two slightly different motifs with homology to the Arabidopsis WUS binding sequence TCA(T/A/G)TCATTCA identified by DAP-seq. WOX proteins often recognize conserved WUS-like motifs, as seen in AtWOX11 binding to the TAAT-like element identified in WUS targets (Lohmann et al. 2001; O’Malley et al. 2016). Unlike soybean and scarlet runner bean WOX9, which bind low-complexity cis-elements (Chen et al. 2021), cis-element variations are likely associated with diverse target genes of WOX9 across plant species.

Our study identified *S*-regulated genes in fruit development, all negatively regulated by *S*, indicating its role as a transcription repressor. The repressive activity of S was further exemplified by its transcriptional repression of *SlTEL1* in seeds. In contrast, in *N. sylvestris* WOX9 acts as an activator in regulation of *NsCKX3* expression in the leaves (Wang et al. 2022b). However, it is unclear *N. sylvestris* WOX9 is a sole activator or bifunctional transcription factor like WUS in Arabidopsis (Ikeda et al. 2009).

It has been hypothesized that WOX9 forms complexes with other transcription factors to regulate its diverse target genes, based on its distinctive binding motifs and a small number of common target genes (Chen et al. 2021). Indeed, yeast two-hybrid screens found AtWOX9 interacts with repressors like SEUSS-like 2 and GIBBERELLIC ACID INSENSITIVE (Arabidopsis Interactome Mapping Consortium 2011; Marín-de la Rosa et al. 2014), providing evidence to support that WOX9 may function as a repressor. However, we can’t rule out the possibility that S acts as a transcriptional activator of genes like *ETX3* and *Solyc05g032620* containing S binding sites, though in distal intergenic regions.

*S* is an early floral transition marker and a key transcription factor that controls inflorescence branching by influencing thousands of genes (Park et al. 2012; Meir et al. 2021). We identified S binding sites in hundreds of genes differentially expressed in the SAMs between wild type and the *s* mutant. Several targets are implicated in vegetative-to-floral transition, including flowering regulators *UF*, *J*, and *SEP3* (Pnueli et al. 1998; Dielen et al. 2004; Huerga-Fernández et al. 2024). Genetic and gene expression analysis show that *UF* activates *S* expression to promote flowering (Meir et al. 2021). Given the *s* mutant has a much weaker flowering phenotype than the *uf* mutant, the plausibility and significance of a feedback regulation on *UF* transcription by S remain to be explored. Nonetheless, the S targets identified here provide a framework for dissecting transcriptional networks governing SAM phase transition.

## Materials and methods

### Plant materials and growth conditions

Seeds of *S. pimpinellifolium* LA1781, LA2706 (Moneymaker) and 2–005 were obtained from the Tomato Genetics Resource Center at University of California, USA (http://tgrc.ucdavis.edu/). The *s-classic* allele from 2–005 was introduced into LA1781 and LA2706 through crossing, and then near isogenic lines were generated by backcrossing to their parental lines for more than five times.

To overexpress the *S* gene, its full-length cDNA was isolated from wild type flowers and fruits by reverse transcription PCR (RT-PCR) using gene-specific primers (primers mentioned here and thereafter can be found in Table S7). The cDNA fragment verified by sequencing was placed in between the *CaMV35S* promoter and the *NOS* terminator of the binary vector *pHX20* (Zhao et al. 2021). For mutating *SlTEL1* using CRISPR-Cas9 approach, guide RNA selection and vector construction was done as described previously (Xu et al. 2019). Briefly, two guide RNAs targeting the first exon were designed using the CRISPR-P online tool (http://cbi.hzau.edu.cn/crispr/). The oligos for the two guide RNAs were cloned into the *psgR-Cas9-At* vector, and then the expression cassette released using *Eco*RI and *Hind*III (New England BioLabs, USA) was cloned into pCAMBIA1300. *S*^*OE*^ (*L104*) and *sltel1-cr*−1 plants in Moneymaker were obtained by backcrossing at least twice before used for phenotypic analysis.

All plants were grown in a phytotron under the following conditions: temperature at 20–25 °C, relative humidity 70–80%, and 150 mE·m–2·s–1 daily light for 16 h.

### Phenotypic observation and measurements

Root lengths were measured on 20–81 seedlings per genotype at five days after germination. 15–21 plants were used for measuring the flowering time as defined by the leaf number below the first inflorescence. Data on fruit weight, seed number, and seed weight were collected from plants grown in the same phytotrons. For plants in Moneymaker background, data were obtained from 8 to 10 fruits per plant and from 9 to 34 plants per genotype. Only plants produced more than five fruits were included for data analysis. For those in LA1781 background, data were collected from five plants for each genotype. Only red ripen fruit were included in the analysis. The seeds extracted from 8–10 fruits were pooled and cleaned with 0.2% hydrochloric acid for 15 min and rinsed with warm water several time before dry under room temperature, then air-dry seeds were weighted. Seed number per fruit was calculated by dividing total seed weight with fruit number and 100-seed weight.

To determine the number and size of pericarp cells, fruit pericarps were harvested, fixed in FAA (10% formalin, 5% acetic acid, 50% alcohol) solution and embedded in Paraplast™ (Sigma-Aldrich, USA). 10 μm sections were obtained using a microtome (Leica, Germany) and stained with a 0.05% (w/v) toluidine blue solution. For leaf mesophyll cell measurements, leaflets of the first true leave at 21 days after germination were dissected and placed in FAA fixative for two hours. Then, leaf segments were cleared using chloral hydrate (Hoyer’s solution) for three hours. After clearing, palisade mesophyll cells were observed under a differential interference contrast microscope. Cell measurements were conducted using the ImageJ software (http://rsbweb.nih.gov/ij/). Each biological replicate was composed of samples from 3–5 plants, and more than thirty cells per section were measured. The experiments were replicated on two batches of plants.

### Transcriptome profiling by RNA-seq and real time quantitative RT-PCR

Total RNA was extracted from roots, stems, cotyledons, leaves, SAM, flower buds and developing fruits with the Trizol reagent (Invitrogen, USA) based on the methods previously described (Xiao et al. 2008).

RNA-seq was conducted on the total RNA extracted from whole fruits of *s*, *S*^*OE*^ (*L104*) and wild type in LA1781 background. For each of the three biological replicates, three to five fruits from five plants per genotype were pooled prior to RNA extraction. Pair-end RNA-seq libraries were generated by using the NEBNext DNA Library Prep Master Mix Set for Illumina (E7420L, NEB) in accordance with the manufacturer’s instructions. Subsequently, sequencing was performed on an Illumina Hiseq2500 machine using Hiseq SBS Kit V3 (Cat # FC-401–3001, Illumina, USA). A previously described workflow for data processing was applied, which included quality control, removal of adapter and low-quality sequences, as well as reads mapping to the reference genome (ITAG version 3.0) (Zhou et al. 2022). Transcript abundance was estimated by using featureCount (http://bioconductor.org/packages/Rsubread) with following parameters: -T 15 -t exon -g gene_id -p –donotsort -C -a ITAG3.2_gene_models.gtf. Then, DEGs (≥ twofold expression change and adjusted *p* < 0.05) were identified by using the DESeq2 software (Anders and Huber 2010). Gene Ontology (GO) enrichment analysis was performed via the online tool PANTHER (Protein ANalysis THrough Evolutionary Relationships) (http://pantherdb.org/).

For qRT-PCR analysis, 5 µg of total RNA with residual genomic DNA removed by RNase-free DNase I set (Cat # 79,254, QIAGEN, Germany) was subjected to cDNA synthesis using the First Strand cDNA Synthesis Kit (Cat # K1621, Thermo Fisher Scientific, USA). qRT-PCR was performed using SYBR® Premix ExTaq™ (Cat # RR390A, Takara Biotech, Japan) on a BIO-RAD C1000 machine (Bio-Rad., USA) or an ABI QuantStudio 3 machine (Thermo Fisher Scientific, USA). Transcript levels were normalized to *SleIF4α6* and reported as relative expression (Xiao et al. 2008).

### In situ hybridization

In situ hybridization was performed on SAMs, flower buds, developing fruits and seeds using digoxigenin-labeled gene-specific probes based on the protocol previously described (Weng et al. 2015). Templates were prepared using following primer pairs: *S* (5’-ATGGCTTCATCAAATAG-3’ and 5’- TTATATATGATGAGTCG-3’, 1077 bp) and *SlTEL1* (5’-ATGGAGAACAATGGTATC-3’ and 5’-CACACAAAGAAGCGAATG-3’, 1048 bp). A T7 promoter sequence was incorporated into each primer for in vitro transcription.

### DAP-seq analysis

DAP-seq protocol was adapted from Bartlett et al (2017) with minor modifications. Genomic DNA was fragmented to 150–200 bp using a Bioruptor Plus machine (Diagenode, Belgium), followed by A-tailing and adapter ligation using the NEXTFLEX-Rapid DNA-Seq Kit (Bioo Scientific Corporation, USA). Post-ligation library purification used the VAHTS DNA Clean Beads (Cat # N411, Vazyme, China).

The full-length S cDNA was cloned into pFN29A (Promega) for in vitro expression of Halo-S fusion protein using the TnT Wheat Germ Extract System (Promega, Cat # L4130). The protein was diluted in 25 μL 1 × PBS and incubated with library DNA for 1 h at room temperature (10 rpm rotation). Magnetic bead-bound protein-DNA complexes were washed six times with 50 μL 1 × PBS, and DNA was eluted in 30 μL Tris–HCl (10 mM, pH 8.5) at 98 °C for 10 min. Eluted DNA was amplified (20 cycles) with Illumina TruSeq primers, purified with VAHTS beads, and sequenced on an Illumina NovaSeq 6000.Raw reads were quality-checked via fastqc (http://www.bioinformatics.babraham.ac.uk/projects/fastqc/) and trimmed with Trimmomatic tool (Bolger et al. 2014) to remove low-quality reads and adaptor sequences. Clean reads were mapped to the tomato reference genome (version ITAG3.2; Sol Genomics Network: solgenomics.net/) using bowtie2 (Langmead et al. 2009) with parameters: -q –no-mixed –no-discordant –no-unal. Unique and mapped reads with mapping quality higher than 30 were used for peak calling. Peaks were called by MACS2 (https://github.com/jsh58/MACS) with input DNA as control, using parameters: -g 7.17e8 –keep-dup 1 -f BAMPE -B -q 0.05. Only peaks within 3 kb upstream of ATG start codons were retained. Motifs enrichment was analyzed by MEME-ChIP (http://meme-suite.org).

### Protein-DNA interaction analysis by EMSA and luciferase assay

To assess S-mediated transcriptional regulation of SlTEL1, a 2,334 bp SlTEL1 promoter fragment was amplified from Moneymaker genomic DNA and cloned upstream of the firefly luciferase (LUC) gene in a reporter vector. The full-length S cDNA was inserted into pHX60 under the 2 × 35S promoter, with empty pHX60 as a control. Agroinfiltration and luciferase activity detection followed our previous protocol (Xu et al. 2019).

EMSA was performed as described previously (Xu et al 2019). S protein was synthesized using the TnT™ SP6 High-Yield Wheat Germ Protein Expression System (Cat #, L3261, Promega, USA) and incubated with biotin- or CY5- labeled probes for 20 min at room temperature. Biotin-labeled probes included P1 (5’-TACTAGAGATTGGTTTTT-3’) and P2 (5’-AATATTTGTTATTAATTTTATCTTTTAGAATATATGATAAAAAGGAATAAATAT-3’) from the *SlTEL1* promoter. DAP-seq-identified S binding motifs were labeled with CY5: Motif 1 (TCAATCATCAATCATCAATCA) and Motif 2 (TCAACGTTCAACGTTCAACGT). Protein-DNA complexes were separated by 4% native PAGE electrophoresis.

### Determination of DNA ploidy of pericarp cells by flow cytometry

DNA ploidy level and Endoreduplication indices (EIs) were determined as described previously (Zhao et al. 2021). Briefly, fruit pericarps from 3–5 plants per genotype were chopped with a razor blade. Then, 100 mg of the chopped pericarps were immersed in 1 ml Galbraith’s extraction buffer (Galbraith et al. 1983) containing 5 mM sodium metabisulfite. Nuclei isolated by passing the suspension through 48-μm nylon mesh twice were stained with 5μg/ml (final concentration) DAPI (4’,6-diamidino-2-phenyindole, Cat # 28,718–90-3, Sigma-Aldrich, USA). DNA ploidy levels were determined by a Beckman flow cytometer (MoFloTM XDP, Beckman Coulter, USA). The experiments were repeated three times using different batches of plants.

## Supplementary Information

Supplementary Material 1: Supplemental Figure S1. Phenotypes of SOE, s and wild type in LA1781 background during vegetative development. Supplemental Figure S2. Phylogenetic tree of SlTEL1 and its homologs in tomato, Arabidopsis, rice and maize.

Supplementary Material 2: Supplemental Tables S1-S7.

## Data Availability

Data and tomato lines generated in the study are available from the correspondence author upon request.

## Abbreviation

*ACL5*: *ACAULIS 5*
*AHG1*: *ABA-hypersensitive germination 1*
*CYL1*: *CYCLOPS 1*
DAP-seq: *DNA affinity purification sequencing*
DPA: *Days post anthesis*
DAPI: *4’,6-diamidino-2-phenyindole*
DEG: *Differentially expressed genes*
EI: *Endoreduplication indices*
ESMA: *Electrophoretic Mobility Shift Assay*
*ETX3*: *EXTENSIN 3*
FAA: *Formalin-acetic acid- alcohol*
GO: *Gene ontology*
*GRP2B*: *Glycine-rich protein 2B*
*JAR1*: *JASMONATE RESISTANT 1*
*J*: *JOINTLESS*
LUC: *Firefly luciferase*
Mei2: *Meiosis protein 2*
*NF-YB6*: *Nuclear transcription factor Y subunit B6*
*NPF7.3*: *NRT1/PTR FAMILY 7.3*
*NsCKX3*: *Nicotiana sylvestris cytokinin oxidase 3*
*PIN2*: *PIN-FORMED 2*
*PME12*: *PECTIN METHYLESTERASE 12*
*PUP4*: *PURINE PERMEASE 4*
qRT-PCR: *Quantitative reverse transcription polymerase chain reaction*
*RGE1*: *RETARDED GROWTH OF EMBRYO 1*
RNA-seq: *RNA sequencing*
S: *COMPOUND INFLORESCENCE*
*S*^*OE*^: *S overexpression*
SAM: *Shoot apical meristem*
*SEP3*: *SEPALLATA3SBT1.8, SUBTILASE 1.8*
*SlTEL1*: *Solanum lycopersicum TERMINAL EAR-LIKE 1*
*SP*: *SELF-PRUNING*
TSS: *Transcription start sites*
*UF*: *UNIFLORA*
WOX9: *WUSCHEL-LIKE HOMEOBOX 9*
*WRI1*: *WRINKLED 1*
WUS: *WUSCHEL*
