# Supplementary material for: Compound Inflorescence (S) represses fruit growth and seed development in tomato
Source: Mol Hortic. 2026 Jan 8;6:5. doi: 10.1186/s43897-025-00183-x (PMC12781540; doi:10.1186/s43897-025-00183-x)
Supplement: Supplementary file 1 — Supplementary Material 1: Supplemental Figure S1. Phenotypes of SOE, s and wild type in LA1781 background during vegetative development. Supplemental Figure S2. Phylogenetic tree of SlTEL1 and its homologs in tomato, Arabidopsis, rice and maize. [file 43897_2025_183_MOESM1_ESM.docx]

**
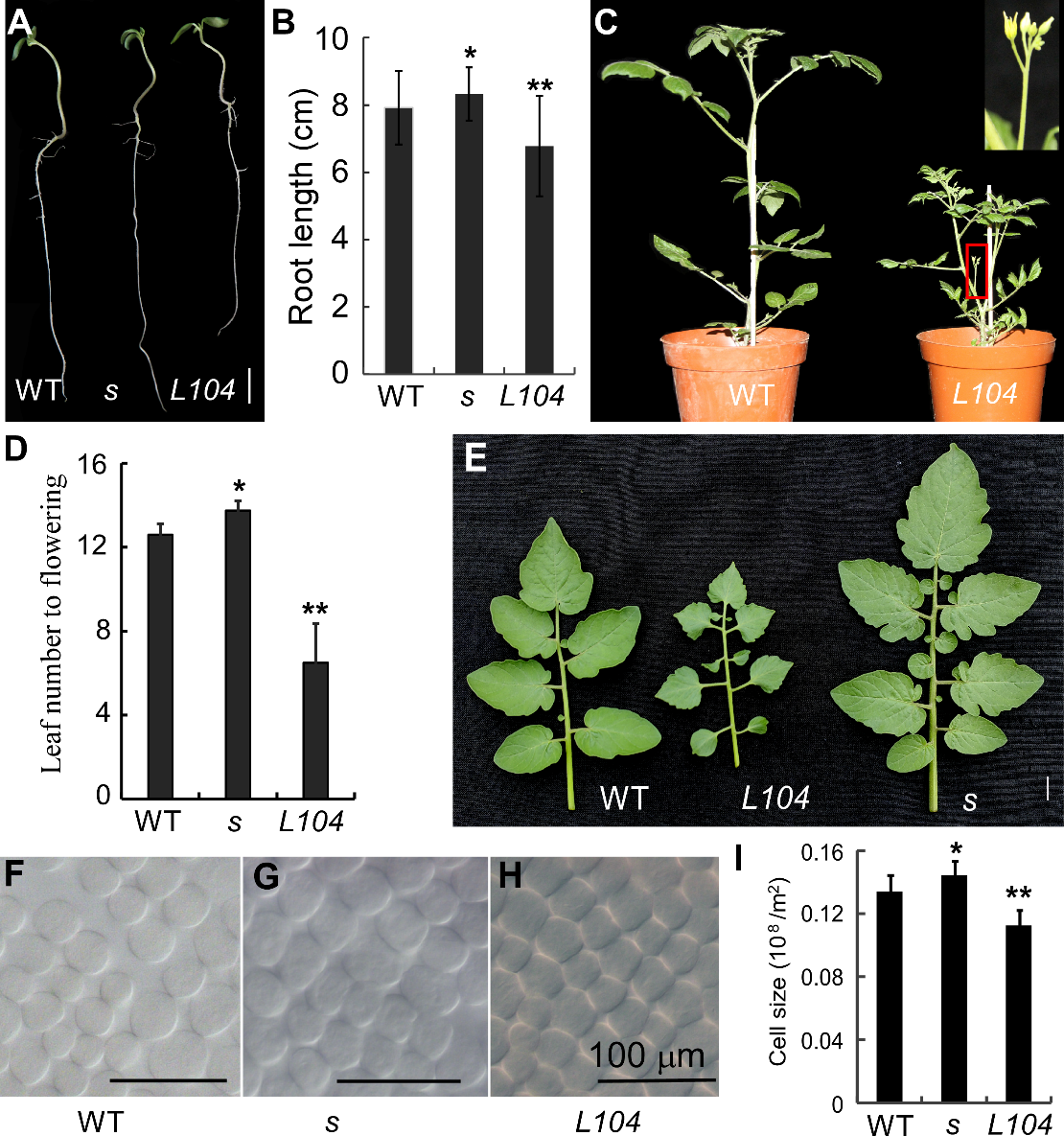
**

**Fig. S1 vegetative morphology of wild type, *s* and *S^OE^* in LA1781 background.**

**A** Root phenotype of wild type, *s* and the *S^OE^* line *L104*. **B** Root length of wild type, *s* and the *S^OE^* line *L104*. **C** Plant stature of wild type and the *S^OE^* line L104. **D** Leaf number before flowering of wild type, *s* and the *S^OE^* line *L104*. **E** Leaf morphology of wild type, *s* and the *S^OE^* line *L104*. **F-H** Cell morphology of wild type (**F**), *s* (**G**) and the *S^OE^* line *L104* (*H*). **I** Leaf epidermic cell size of wild type, *s* and the *S^OE^* line *L104*. Statistical significance was based on Student’s t-test. *, *p* <0.05; **, *p* < 0.01. Scale bars represent 1 cm in (**A, E**) and 100 μm in (**F-H**).

**
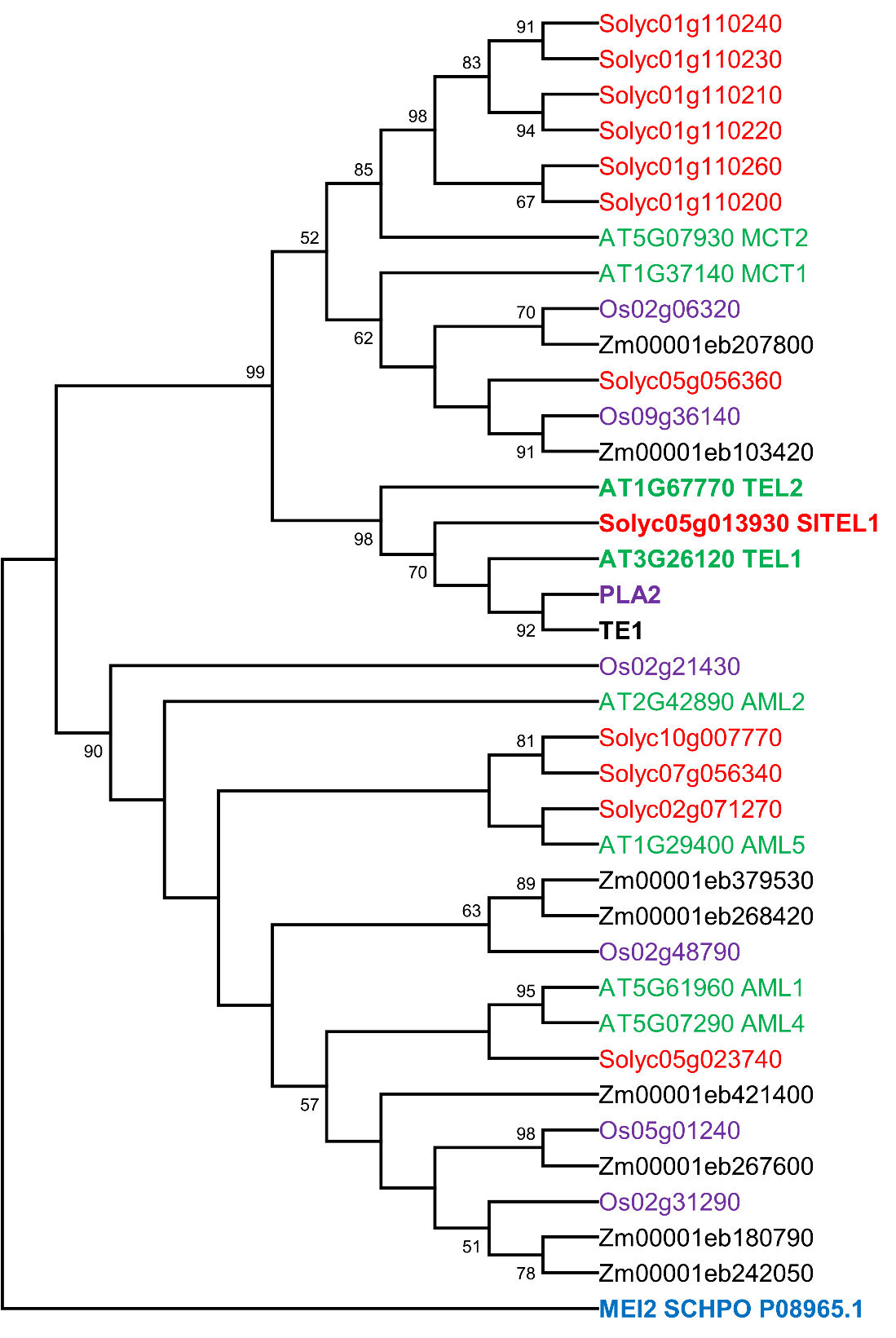
**

**Fig. S2 Phylogenetic analysis of TEL proteins.**

The phylogenetic analysis was performed by using MEGA7 (Kumar S., Stecher G., and Tamura K. (2016) MEGA7: Molecular evolutionary genetics analysis version 7.0 for bigger datasets. Mol Biol Evol 33:1870-1874). Maximum Likelihood method was used, the percentage of trees (only those higher than 50% are shown here) in which the associated proteins clustered together is shown next to the branches.
